# Examining the usability and viability of using a simulated classroom environment to prepare preservice science teachers during and after the COVID-19 pandemic

**DOI:** 10.1186/s43031-022-00054-1

**Published:** 2022-06-15

**Authors:** Jamie N. Mikeska, Heather Howell, Devon Kinsey

**Affiliations:** ETS Educational Testing Service, 660 Rosedale Rd, Princeton, NJ 08541 USA

**Keywords:** Argumentation, Discussions, Elementary, Practice-based teacher education, Science, Simulated teaching

## Abstract

Educator preparation programs experienced extreme challenges during the COVID-19 pandemic, as many universities and K-12 schools moved to fully online or hybrid instructional models. These abrupt changes significantly limited preservice teachers’ opportunities to engage in classroom-based practice teaching experiences, which are a bedrock of educator preparation programs to support preservice teachers in learning how to teach effectively. In this study, we examined the usability and viability of integrating simulated teaching experiences, which occur in an online, virtual classroom environment consisting of five student avatars, into elementary science method courses during the COVID-19 pandemic to prepare preservice science teachers to engage in one critical science teaching practice: facilitating discussions that engage students in scientific argumentation. This study uses qualitative content analysis of survey data and a focus group interview to identify patterns and themes in how four elementary science teacher educators and 49 of their preservice teachers perceived the use of this tool within elementary science teacher education, particularly the opportunities and challenges this tool afforded during the pandemic and possibilities for use in the post-COVID era. Study findings suggest that these elementary science teacher educators and preservice teachers perceived the simulated teaching experience as valuable for supporting teacher learning, addressing COVID-related challenges, and tackling perennial challenges in science teacher education. They also noted challenges related to implementation and concerns with future access. A discussion of key factors that may support and hinder the use of such tools within elementary science teacher education and implications for leveraging lessons learned post-COVID are included.

## Introduction

The Next Generation Science Standards (NGSS) has redefined K-12 science instruction to focus on a three-dimensional model of learning that attends to students’ integrated understanding and use of disciplinary core ideas, cross-cutting concepts, and scientific and engineering practices (National Research Council, 2011; NGSS Lead States, 2013). This new vision for science instruction has required considerable reconceptualization of learning opportunities in science teacher education so that science teachers can successfully enact this NGSS-aligned vision in their classrooms (Reiser, 2013). One perennial challenge in science teacher education has been how to provide substantive, practice-based learning opportunities for preservice teachers (PSTs) to tryout and learn how to engage in novel, complex teaching practices aligned with this new instructional vision. While field experiences have played a major role in providing these learning opportunities, that has not always been possible in elementary science due to limited instruction in this content area at the elementary level (Blank, 2013). And in the recent case of the COVID-19 pandemic, these practice-based teaching opportunities were significantly limited or non-existent as universities and K-12 schools moved to fully online or hybrid instructional models (Reich et al., 2020; Saenz-Armstrong, 2020).

To address this perennial challenge, elementary science teacher educators (TEs) have turned to other practice-based approaches to provide these learning opportunities (Benedict et al., 2016), including the use of micro-teaching, peer rehearsals and video-based analysis of elementary science instruction (Benedict-Chambers et al., 2020; Davis et al., 2017; Masters, 2020; Roth et al., 2018). More recently, novel approaches offer such practice-based experiences on a digital platform using a virtual classroom environment with student avatars and have the advantage of being able to be conducted online remotely (Lee et al., 2018; Mikeska & Howell, 2020; Mikeska, Howell, Dieker, & Hynes, 2021; Straub et al., 2015). However, the field has a limited understanding of the affordances and challenges of using and integrating such tools within teacher education settings, especially in specific content areas like science. Currently there is a need for research to understand the usability and viability of simulated teaching experiences, especially for PST populations that tend to have limited opportunities to engage in practice teaching in science classrooms. This need has been even more pronounced during the recent COVID-19 pandemic that resulted in the shuttering of K-12 schools and universities and limited opportunities for PSTs to practice teaching.

## Research focus

This study’s research questions focused on examining the usability and viability of integrating simulated teaching experiences into elementary science method courses that are delivered via fully online or hybrid instructional approaches. Previous research has investigated similar questions but in the context of face-to-face instruction within elementary science and mathematics methods courses (Mikeska & Howell, 2021b). This research extends previous research by examining the use of this tool within online or hybrid learning contexts to determine use and application in a wider diversity of settings.

By usability we mean understanding how well TEs and PSTs perceive they can use the simulated teaching experience to achieve key learning goals effectively and satisfactorily. By viability we refer to the capacity of the simulated teaching experience to be sustained over time – in this case, both during and after the COVID-19 pandemic – as part of elementary science teacher education. We addressed these aspects using the following research questions on PST use (research question 1) and TE integration (research question 2): (a) Research Question 1: What do the PSTs perceive they learned from the simulated teaching experience? What are their perceptions of challenges they faced during the simulated teaching experience?; (b) Research Question 2: How do TEs perceive the affordances and challenges of integrating simulated teaching experiences within elementary science method courses? How do they envision using this tool post-COVID to support PST learning?

## Background

### The role of teaching practicum experiences in supporting teacher learning

Student teaching, also referred to as clinical experience, fieldwork, or internship, has been a staple of teacher preparation for decades (Zeichner, 2012), with every state in the United States currently requiring some form of clinical experience in order to receive a teaching certificate via a traditional pathway. The reasons for assuming such practice is needed are so widely accepted that they are rarely articulated, including rationales for supporting teacher learning as well as gatekeeping for the profession. Novice teachers need to learn to do the work of teaching and engaging in teaching is a method for doing so. In addition, it is assumed that novice teachers should first teach under the watchful eye of someone more experienced to ensure that they are adequately prepared. Thus, student teaching serves multiple roles, allowing the novice to work with and be mentored with a more experienced colleague, to enact pedagogy they are learning about in coursework, and to be judged as to their readiness to take on classroom instruction independently (Korthagen et al., 2006). These opportunities tend to be impactful. Research has shown that novice teachers cite student teaching opportunities as the most important component of their preparation (Levine, 2006) and studies find that the quality of mentoring matters, suggesting the power of a strong practicum experience (Goldhaber et al., 2020).

Yet, practice in schools, while acknowledged as a critical step in teacher preparation, has limitations, particularly when it constitutes the only way of practicing available to aspiring teachers. The same evidence that suggests that high quality mentoring matters suggests that lower quality mentoring could be problematic, and student teaching placements with appropriate mentors can be difficult to come by (Greenberg et al., 2011). Student teaching, because it initiates the novice into an existing system, can also reinforce the replication of elements of that system, and combined with novice teachers’ prior experience as students within such systems can make enactment of new and different teaching approaches quite difficult (Gray, 2020; Lortie, 1975). Student teaching also demands of novices that they enact a complete version of teaching. While they may do so with support, there is little opportunity for gradual scaffolding. Finally, student teaching sometimes takes place as a relatively separate component of university preparation and is not always well integrated with coursework (Allen & Wright, 2014), although there have been many notable attempts to strengthen these connections (Zeichner, 2010; Zenkov et al., 2019). This feature of student teaching leaves the novice teacher to attempt to make connections between academic learning and practical application independently.

The field has proposed several approaches to help mitigate these limitations and better bridge the space between academic learning and student teaching, most recently in the associated movements referred to as Practice-Based Teacher Education (PBTE) and Core Practices. PBTE is an umbrella term used to describe programmatic shifts in teacher preparation in which teaching practice is integrated into learning earlier, more frequently, and in more scaffolded ways (Ball & Forzani, 2009; Grossman et al., 2009). The idea is to engage novice teachers in the work of teaching before they enter the classroom with students, and to allow them to practice discrete components of the work of teaching in meaningful ways with support. The closely aligned Core Practices movement suggests a differentiation among teaching practices that identifies some as more critical for teachers to master, allowing programs of teacher education to focus more narrowly on what will be most useful (Forzani, 2014; McDonald et al., 2013). Taken together, these frameworks suggest that, prior to working with K-12 students, novice teachers should spend time practicing the most critical teaching practices, or components of them, as part of university coursework and as part of a structured trajectory of teacher learning.

What PBTE looks like can vary. Grossman et al. (2009) discusses three pedagogies of enactment – representations, decompositions and approximations – as a lens for engaging novices in teaching practice. Representations can include activities such as watching videos of classroom teaching to practice noticing salient features of instruction (Wenner & Kittleson, 2018). Decomposition is the work of identifying the core components of teaching, each of which is more manageable to learn and can then be recomposed into robust instruction (Grossman et al., 2009). Approximations are activities that engage the novice in doing some part of the work of teaching in a context of limited complexity, such as rehearsing an instructional sequence with peers or responding to a case study. Research has shown that TEs’ use of practice-based teaching approaches, like the use of approximations, can be challenging to enact productively, although they do afford learning opportunities that differ when using other teaching pedagogies (Peercy & Troyan, 2017). In this study, we focus on examining the use of one tool – an online, simulated classroom consisting of five upper elementary student avatars – to engage PSTs in approximations of one core teaching practice that is critical to science teaching and learning: facilitating discussions that engage students in scientific argumentation.

### Learning how to engage students in scientific argumentation

Engaging in argumentation from evidence is one of the key scientific practices important for students to develop their understanding of scientific phenomenon and support their scientific sensemaking (NGSS Lead States, 2013). Scientific argumentation involves students in two key processes – argument construction and argument critique – which map onto the structural and dialogic aspects of this practice (Gonzalez-Howard & McNeill, 2019; Grooms et al., 2018; Jiménez-Aleixandre & Erduran, 2008). Productive argument construction provides opportunities for K-12 students to generate evidence-based scientific claims, justify those claims, consider competing claims, and revise their claims based on new evidence and reasoning (McNeill et al., 2016). Argument critique targets the dialogic aspect of scientific argumentation where students evaluate one another’s evidence-based reasoning, offer counterarguments and rebuttals, and consider the adequacy and relevancy of scientific evidence (Kuhn, 2010; McNeill et al., 2016; Osborne et al., 2013). Together these two aspects of argumentation complement each other as students collectively work toward coming to consensus on their explanations of scientific phenomenon.

While there are varied approaches that K-12 science teachers can use to engage their students in scientific argumentation, one of the most frequently used and well-known approaches involves the use of whole or small group discussions (National Research Council, 2011). Facilitating argumentation-focused science discussions is a hallmark of high-quality science instruction and is noted in the science teaching standards as an approach that provides a supportive context for students to engage in scientific sensemaking (National Science Teachers Association, 2012). Most importantly, research has suggested that engagement in such discussions can support students in developing their conceptual understanding, their thinking processes, and their epistemic practices (Cartier et al., 2013; Chinn, 2006; Duschl & Osborne, 2002). Research has indicated that many factors impact students’ and teachers’ abilities to, respectively, engage in or facilitate science discussions including their conceptual understanding, their previous experiences with discussions, the classroom and curricular contexts in which the discussions are used, and perceptions about teachers’ or students’ self-efficacy and capabilities (Colley & Windschitl, 2016; McNeill et al., 2016, 2017; Osborne et al., 2013; Sadler, 2006).

Research has suggested that learning how to engage students in productive scientific argumentation requires structured and scaffolded learning opportunities. There have been a few notable efforts in the last decade to develop and use targeted resources and supports, including professional development programs, to support K-12 teachers in learning how to engage students in scientific argumentation. Two particularly robust examples stand out. First, researchers and science teacher educators on the Argumentation Toolkit project (www.argumentationtoolkit.org) developed a suite of multimedia modules to help science teachers learn about how to plan for and engage their students in argumentation. As part of these learning modules, teachers had opportunities to build their understanding of the different elements of argumentation (e.g., claims, evidence, reasoning), analyze students’ engagement in scientific argumentation through various written and video examples, and plan argumentation-focused science lessons (Marco-Bujosa et al., 2017; McNeill et al., 2016, 2018). They found that teachers who engaged in learning with these multimedia materials improved their self-efficacy, their ability to generate and attend to student learning goals, and their perceptions about students’ abilities. More recently, a team of university researchers collaborated on a project in which they developed a multi-year professional development (PD) program, which they coined the PRACTISE PD model, where practicing teachers engaged in an institute, practicum, and follow up sessions throughout the school year to learn how to support argumentative discourse between students (Fishman et al., 2017; Osborne et al., 2019). In the practicum component, teachers practiced facilitating argumentative discourse with their peers. Results from this study showed that there was improvement in both teacher and student discourse practices across three different versions of the PD program, and these positive changes were sustained across multiple years. This study extends the focus of previous research in this area by examining the usability and viability of an innovative technological tool – a simulated classroom – for supporting PSTs to engage in approximations of practice to learn how to facilitate argumentation-focused discussions in elementary science. If findings support the tool’s usability and viability within online and hybrid teacher education courses in this context, then this tool could broaden the resources that TEs have at their disposal to support PSTs in building expertise in this core teaching practice – and possibly others – within science and across other disciplines.

## Methods

This study used qualitative content analysis (Schreier, 2014) to examine the TEs’ and PSTs’ perceptions on the usability and viability of integrating simulated teaching experiences into elementary science method courses. This analysis approach supports the interpretation of meaning from written and spoken text and, as such, provided a way to identify patterns or themes across the survey and interview responses to address the study’s research questions.

### Sample

Participant recruitment began by sharing information with elementary TEs about the study via the listservs or blog posts of professional organizations, such as the Association of Science Teacher Educators (ASTE), the National Association for Research in Science Teaching (NARST), and the American Association of Colleges for Teacher Education (AACTE). Project leads also sent individual communications to faculty who had submitted letters of collaboration as part of the grant submission process. Interested participants responded to an online survey providing information about their institution’s characteristics, why they were interested in participating in the study, and their qualifications for study participation, including information about the course they planned to integrate the simulated teaching experience into, the characteristics of the college students who typically enroll in this course, and the current challenges they were facing due to the COVID-19 pandemic.

In total, we had 95 elementary TEs complete the full online application survey.^1^ Based on the recommendations from our project’s advisory board, we prioritized selection of TEs who were working at a minority serving institution or a historically black college or university and those who were working at a university with a larger percentage of black or Hispanic college students enrolled. We also considered the urgency of the challenges the TEs were facing due to the COVID-19 pandemic and whether their university policies would allow them and their PSTs to participate in this study. After selecting the TEs for study participation, we then used a nested recruitment method to recruit the PSTs for study participation. Every PST in the course in which the TE was planning to integrate the simulated teaching experience into was provided an opportunity to consent to participate in this research study.

Four elementary science TEs (one male, three female) participated in this research study and integrated the simulated teaching experience into one of their elementary science method course sections in fall 2020 at their university (three public and one private institution; three urban and one suburban location). Due to the COVID-19 pandemic, two courses were taught in an entirely online format while the other two courses were taught via a hybrid format using both online and face-to-face instruction. All four courses were taught synchronously. Forty-nine of the PSTs (three males, 46 female) across these four elementary methods sections participated in the study.

The participating PSTs’ reported ethnicity was diverse with 47% Hispanic/Latino, 12% Black, 4% Asian, 41% Caucasian, and 4% Other. Two PSTs (4%) reported previously obtaining a bachelor’s degree—one PST had a major of Elementary Education and the other PST did not specify their undergraduate major. However, no PSTs reported previously obtaining an advanced degree. The PSTs reported their majors in their current program as Elementary Education (55%), Early Childhood Education (51%), Special Education (12%), Bilingual Education (6%), Secondary Education (4%), English Language Arts (4%), Foreign Language (2%), Math/Computer Science (2%), Natural Sciences (2%), and Social Sciences (2%), with many selecting multiple current majors. Although most of the PSTs (65%) had previously completed a science content course at the college level (e.g., college biology, physics, earth science, engineering), only a small proportion of PSTs had already completed a science methods/pedagogy course (12%) or a science content course designed specifically for K-12 teachers (12%). Most PSTs (82%) and their TEs (three of the four) reported having no prior experience using simulated classrooms. Table 1 provides details about the study participants’ background.

**Table 1:** Elementary Science Teacher Educators and Preservice Teacher Characteristics

Characteristics		Teacher Educators (*n* = 4) n	Preservice Teachers (*n* = 49) n (%)
Gender	Male	1	3 (6%)
	Female	3	46 (94%)
Ethnicity^a^	Hispanic/Latino	0	23 (47%)
	Black or African American	0	6 (12%)
	Asian or Asian American	0	2 (4%)
	Caucasian	3	20 (41%)
	Other	0	2 (4%)
Institution Type	Public	3	43 (88%)
	Private	1	6 (12%)
Institution Location	Urban	3	37 (75%)
	Suburban	1	12 (25%)
	Rural	0	0 (0%)
Course Format	Online Only	2	31 (63%)
	Face-to-Face Only	0	0 (0%)
	Hybrid	2	18 (37%)
Course Structure	Synchronous Only	4	49 (100%)
	Asynchronous Only	0	0 (0%)
	Hybrid	0	0 (0%)
Prior Experience Using Simulations	Yes	1	9 (18%)
	No	3	40 (82%)

ªOne teacher educator did not respond to this question. Totals do not add to 100% because each participant could make one or more selections for ethnicity

### Data collection

**Fig. 1:**
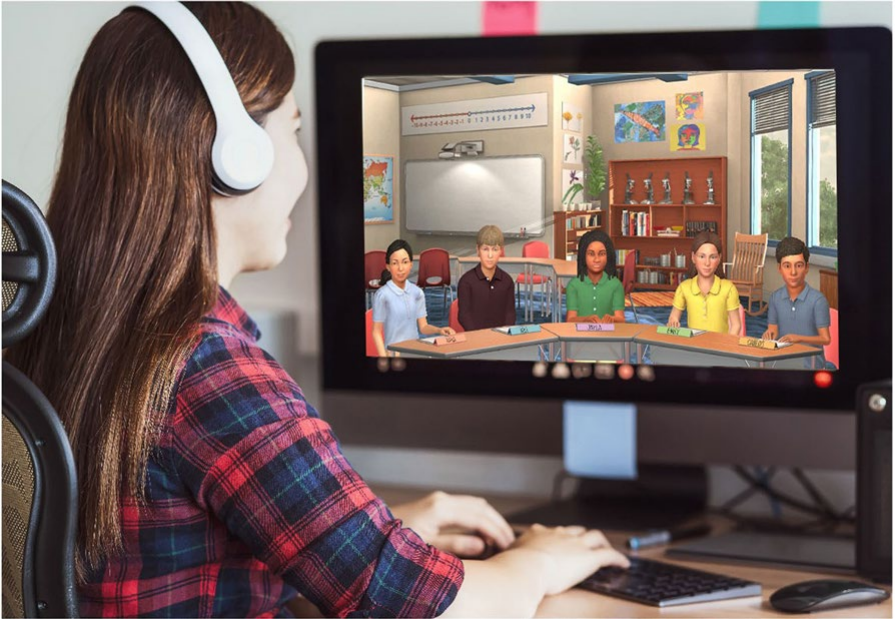
Preservice Teacher Interacting with Student Avatars in the Simulated Classroom. Image courtesy of Mursion, Inc.

Each PST in this study participated in the simulated teaching experience as part of their elementary science methods course in fall 2020. The simulated teaching experience consisted of three main components: (a) preparing for facilitating a science discussion in the simulated classroom, (b) facilitating a discussion in the simulated classroom on a given science topic, and (c) debriefing and reflecting on the discussion afterwards. Each TE decided how to help their PSTs prepare for (first component) and debrief/reflect on (third component) their simulated discussion as part of their coursework (Mikeska, Webb, et al., 2021). During the second component, each PST facilitated a discussion on their own on the given science topic using the *Mystery Powder* science performance task (Mikeska, Howell, Ciofalo, et al., 2021a) for up to 20 min in the simulated classroom. The simulated classroom (see Fig. 1) consists of five upper elementary student avatars, who are controlled on the backend by a human-in-the-loop called an interactor. The interactor uses specialized technology to sound like and respond as fifth grade students during the discussion. This simulated classroom was developed as part of an earlier project (National Science Foundation grant #1621344) in collaboration with the project’s technology partner, Mursion, Inc. The design of the simulated classroom and five student avatars was conducted with input from relevant stakeholders with expertise in elementary instruction, educational research, and technology. Certain decisions, such as the number of student avatars, were directly related to the capacity of the technology available for use at the time of this study. Each simulated discussion occurred outside of the PSTs’ class time and was conducted individually with each PST without the TE or any other observers present.

The *Mystery Powder* performance task was developed on an earlier project (Mikeska et al., 2019; Mikeska, Howell, Ciofalo, et al., 2021a). This task is a written packet that our research team provided to each PST approximately one week prior to their scheduled discussion in the simulated classroom. The packet provides the PST with information about the student learning goal for the discussion and what prior experiences and classroom activities the students have completed before the discussion. It also gives the PST information about where this discussion fits into the larger instructional sequence and science lesson. In addition, the packet provides the PST with copies of the students’ written work to use in their planning and gives them access to example video clips so the PST can learn about teaching moves to engage students in argumentation-focused science discussions. These video clips are part of the Argumentation Toolkit website, which is an online resource that includes a set of interactive tools and videos to help teachers build their understanding about and ability to teach scientific argumentation.^2^ These video clips illustrate the various ways that science teachers have integrated argumentation into their science instruction.

The goal of the My*stery Powder* science discussion is for the PST to support the students in coming to consensus, using evidence and reasoning, on the identity on an unknown mystery powder and which properties are most useful to identify the mystery powder. Prior to the discussion, the students in this class had collected data on various properties (e.g., color, texture, reaction with vinegar, etc.) of six known powders (e.g., sugar, salt, baking soda, etc.) and one unknown mystery powder and had completed an entry in their science journal explaining their claim and evidence-based reasoning about the mystery powder’s identity. The five students begin the discussion with varying claims and evidence. While the PSTs were expected to read through and use the *Mystery Powder* task packet in their preparation, each of the TEs also engaged the PSTs in one or more preparation activities to support them in preparing for the simulated discussion. Therefore, the task packet itself was not the only support or scaffold provided to the PSTs prior to facilitating the discussion. The PSTs could also decide what to do and how to prepare for their simulated discussion using the task packet and the preparation activities facilitated by their TE.

A few notes about how the simulated discussion works are important to highlight. First, prior to the discussion, the interactor (the person behind the scenes) completes extensive training to learn how to act and respond as the five upper elementary student avatars in the simulated classroom and prepare for the science discussion. This training, which takes approximately 60 to 80 h in total, is multifaceted and includes a series of synchronous and asynchronous training sessions led by one or more trainers. In particular, the training involves the interactor in learning:

- how to use the equipment to sound and move like upper elementary students,
- how to use the online portal developed by Mursion, Inc. to launch, engage in, and video record the simulated discussion session,
- about the students’ personal backgrounds,
- about the students’ initial claims and evidence-based reasoning from the *Mystery Powder* task and science content related to this task that could come up during the discussion,
- when and how the students should “learn” during the discussion and revise their initial arguments about the mystery powder’s identity and which properties are useful to determining what the mystery powder is (as needed), and
- how students should respond to each other and the teacher during the discussion, including when they should or should not interact directly with each other.

Second, the interactor is trained to provide the PSTs with a *semi*-*standardized* learning experience wherein each PST has the opportunity to facilitate the discussion with the same five student avatars who begin the discussion with the same initial ideas and previous learning experiences. These interactions are not scripted; rather the interactor’s training is grounded in representing the student ideas in consistent and research-backed ways within an improvisational frame that allows for responsiveness and change. This feature means that each PST encounters the same initial teaching challenge. For example, Mina and Will begin the discussion with an incorrect claim (they think the mystery powder is flour) and insufficient and inaccurate evidence-based reasoning. In addition, two other students (Jayla and Emily) start the discussion with an accurate claim (that the mystery powder is baking soda) but use irrelevant evidence to justify their claim. Irrespective of the PST facilitating the discussion, the same students start with the same claims and evidence-based reasoning, which results in each PST having to contend with similar challenges and determine how to help these students recognize gaps in their approach and thinking.

While the starting points are the same for each PST’s discussion, the way in which the discussion progresses is not standardized, and no two discussions, facilitated by different PSTs, are the same. Instead, the interactor is trained to follow the PSTs’ lead and let each PST decide what approach they want to take in their discussion facilitation. For example, if a PST makes teaching moves to facilitate a teacher-directed discussion where students are asked to raise their hands to speak and are rarely encouraged to share their reasoning, then the student avatars in that discussion will wait to be called on to answer any of the teachers’ questions and will only respond to the questions asked by the PST. Alternatively, if a PST encourages the student avatars to justify their claims, offer rebuttals in response to their peers’ ideas, persuade each other, and speak to each other directly, then the student avatars will engage in those behaviors during the discussion in response to the PST’s prompts. Thus, the interactor is trained to provide each PST with a semi-standardized learning experience that flexibly adjusts to the teaching approach each PST uses to facilitate the science discussion. As a result, the actual discussions that the PSTs facilitate do not operate like a script but operate in the context of a carefully crafted set of previously designed parameters that unfold in response to the PST’s facilitation, reflecting adaptive situations that occur in real classrooms. Earlier research (Mikeska & Howell, 2021a) has shown that PSTs and TEs perceive these kinds of tasks, including the *Mystery Powder* task, as authentic and reasonable representations of what elementary teachers are asked to do in the classroom. A more extensive discussion of the design and delivery of these semi-standardized performance tasks can be found in Mikeska et al. (2019) and access to the full set of interactor training materials for the *Mystery Powder* science task can be found in Mikeska, Howell, Ciofalo, et al. (2021b).

Our research team collected various data throughout the simulated teaching experience, although the analysis reported in this manuscript uses results from the background questionnaires, the PST and TE surveys, and the TE focus group interview only. First, each TE and PST participant completed a short, online background questionnaire about their demographics, teaching experience, and educational background. For the TEs, this questionnaire asked them to provide information on the elementary teacher education program where they currently teach (e.g., location, types of teachers they work with, etc.) and information on the course that they planned to integrate the simulated teaching experience into. For the PSTs, the questionnaire asked them to provide information about their previous and current degrees and major area(s) of study and the types of courses they have already taken.

Second, the first author attended a few of the TEs’ online class sessions to observe the preparation and debrief/reflection activities that the TEs facilitated in their course prior to and after the simulated discussions and collected any class assignments and artifacts related to the simulated teaching experience. These observations included different types of class activities, such as observing the PSTs watching videos (their own or others) and discussing what they noticed, discussing key features of high-quality science discussions, making sense of data from the *Mystery Powder* science task, or sharing their reflections about the discussion experience with each other. The purpose of conducting these observations and collecting these assignments and artifacts was to gather data on the types and nature of the preparation and debrief/reflection activities that the TEs used to support the PSTs in preparing for and learning from the simulated discussion, as well as to understand how the PSTs responded to these activities, including any challenges they encountered. Outcomes related to the TEs’ use of various preparation and debrief/reflection activities to support PST learning can be found in Mikeska, Howell, and Kinsey (2022).

Third, following the simulated teaching cycle (preparation + simulated discussion + debrief/reflection), each PST completed an online survey about their experience. In this survey, the PSTs reported on several different aspects, such as their main takeaway or learning from this experience, their reported success of meeting the discussion goal and various discussion elements, their perceptions on the preparation and debrief/reflection activities, and their reflections on their overall discussion performance. To examine what the PSTs perceived they learned from the simulated teaching experience, we analyzed their written responses to the following open-ended survey question.

PST survey question 25: Briefly describe your main takeaway or learning from the simulated discussion teaching activity cycle (including the preparation, discussion in the simulated classroom, debrief/reflection, and any other course assignments or activities related to this cycle) this semester.

To better understand their perceptions of challenges they faced during the simulated teaching experience, each PST first responded to a Likert scale question where they selected whether they agree, somewhat agree, somewhat disagree, or disagree with the following statement.

PST survey question 15: I did not perform as well as I could have.

For any PST who selected agree or somewhat agree with that statement, they also provided a written response to this open-ended survey question.

PST survey question 17: Please explain why you did not perform as well as you could have in facilitating the discussion.

Our goal in analyzing the PSTs’ responses to this question was to explicitly look for evidence that the nature of the simulation constituted a significant impediment to PST performance or learning, which would indicate challenges related to the usability of the simulated teaching experience to achieve key learning goals.

Fourth, each TE completed an online survey and participated in a focus group interview after the simulated teaching cycle was complete. In their survey and interview responses, the TEs provided their perceptions on several aspects, such as what their PSTs learned, the adequacy of the preparation and debrief/reflection activities they used, the affordances and challenges they experienced when using the simulated teaching experience, and their overall impression of the feasibility and usefulness of this approach. To understand the TEs’ perspective on what their PSTs learned from the simulated teaching experience, we analyzed their written responses to the following open-ended survey question.

TE survey question 32: What are the big takeaways your PSETs [preservice elementary teachers] learned from participating in the simulated teaching experience (including the preparation, discussion in the simulated classroom, and debrief/reflection)?

To identify patterns in the key affordances and challenges the TEs encountered when integrating the simulated teaching experience into their course during the pandemic and to understand how the TEs were envisioning the use of this tool post-COVID, we analyzed their responses across one survey question and multiple focus group interview questions.

TE survey question 38: What would be the impediments to incorporating simulated classroom experiences into a future course?

TE focus group interview question 14: In what ways, if at all, did this simulated teaching experience support you in addressing specific COVID-related challenges that you, your institution, and/or your preservice teachers faced this past semester?

TE focus group interview question 15: What affordances, if any, were there in terms of incorporating the simulated teaching experience in your course?

TE focus group interview question 16: What challenges did you encounter when incorporating the simulated teaching experience in your course?

TE focus group interview question 18: How would you plan differently if you were to use the simulated teaching experience again in a future course?

TE focus group interview question 19: Overall, what do you think about incorporating simulated teaching experiences, like the one that your preservice teachers experienced, into elementary science methods courses?

Prior to their use, the project’s advisory board members conducted a content review of the questions on both surveys. In addition, similar survey questions were used on a previous study and analysis of responses indicated they were adequately capturing participants’ perceptions on these aspects.

### Data analysis

To examine the PSTs’ perceptions of the simulated teaching experience and the TEs’ use of this approach to promote PST learning, we used a two-pronged approach. First, for Likert-scale survey questions, we used frequencies and descriptive statistics to identify trends. Second, we used a general qualitative analysis approach (Maxwell, 2013) to examine the survey and interview data to identify themes and patterns in the participants’ responses. Specifically, we generated coding schemes to examine what the PSTs reported learning from the simulated teaching experience, what challenges the PSTs and TEs faced, the opportunities this tool afforded during the pandemic, and possibilities for use in the post-pandemic era. After developing and refining the codes, two research team members double coded approximately 20% of the data and reconciled any disagreements, as needed.

For each round of coding, we followed a set of process steps, allowing for satisfactory reliability. To develop the codes, one team member initially skimmed through the responses of each item to identify common ideas. The team member then drafted the descriptions of each code and pulled example responses to be used in the codebook. The team member then presented the codebook descriptions to the other two team members who provided suggestions for code revisions. Once the team finished the codebook review the double coding process varied depending on whether the item being coded was from the PST survey or the TE survey. For the PST survey, approximately 25% of the data within each of the four sites was identified to be separately coded by two team members. The coding from both team members was then reconciled to address any areas of disagreement. Those codes were compared and used to determine interrater reliability. Finally, the remaining 75% of responses were coded by one coder. For the TE survey, since there was a maximum of four responses per item, 100% of the data was doubled coded by two team members who followed the same reconciliation process. Again, this coding was used to determine interrater reliability.

Overall rater agreement for each PST and TE task survey item was within acceptable ranges, including PSTs’ main takeaway or learning [94% exact agreement; 0.83 intraclass correlation coefficient (ICC)], PSTs’ reason for not performing as well as they could have (94% exact agreement; 0.84 ICC), and TEs’ impediments to using simulated teaching experiences in the future (93% exact agreement; 0.91 ICC). After coding the PST and TE task survey responses for each question, we then calculated the number and percentage of responses representing each code and compared coding frequency to identify patterns.

## Results

### PSTs’ perspectives on using simulated teaching experiences (PST use)

#### Main takeaway/learning

Table 2 shows the codes applied to characterize the PSTs’ perceptions about the main takeaways from the simulated teaching experience. About a third (31%) of the PSTs noted that this experience helped them recognize the importance of adequately and thoroughly preparing for facilitating these kinds of discussions with students. For many of the PSTs in this study, this was the first time that they had an opportunity to facilitate a science discussion with students. Recognizing the importance of making sure that they had specific questions and prompts ready for use, as well as an overall outline of the discussion plan linked to the student learning goal, was an important takeaway from this experience. For example, PST 810’s main takeaway was, “…that preparation is key. I didn’t start looking at the simulation materials until an hour before my scheduled time, and if I would’ve looked at them more in advance, I would have realized I had a lot of information to take in before starting my simulation.”

**Table 2:** Coding for Perceptions of Learning from the Simulated Teaching Experience

Code	Description for Perceptions
Student Participation (Dimension 1)	Describes a main takeaway or learning from the simulated discussion teaching activity cycle linked to being responsive to students, focusing on making sure that the discussion is grounded in students’ ideas, and/or that all students are engaged in some meaningful component of the discussion.
Discussion Structure (Dimension 2)	Describes a main takeaway or learning from the simulated discussion teaching activity cycle linked to the degree to which the teacher is able to shape a coherent discussion and/or focuses in particular on building and connecting ideas toward a learning goal.
Student Interactions (Dimension 3)	Describes a main takeaway or learning from the simulated discussion teaching activity cycle linked to the ways that teachers strive to facilitate the discussion so that students are responsible for explaining key ideas to each other.
Student Understanding (Dimension 4)	Describes a main takeaway or learning from the simulated discussion teaching activity cycle linked to the extent to which the teacher and students are involved in the evaluation of ideas that are put forth during the discussion and/or focusing on helping students to build their conceptual understanding.
Argumentation (Dimension 5)	Describes a main takeaway or learning from the simulated discussion teaching activity cycle linked to the degree to which students engage in argumentation.
Preparation Importance	Describes a main takeaway or learning from the simulated discussion teaching activity cycle linked to a general importance of preparing for the discussion.
Flexible	Describes a main takeaway or learning from the simulated discussion teaching activity cycle linked to being flexible when preparing for and/or when leading the discussion.
Recognize Discussion Importance/Challenge	Describes a main takeaway or learning from the simulated discussion teaching activity cycle linked to the importance and/or challenge of including discussion in a math/science classroom.
Importance of Preservice Teachers’ Emotional Regulation as Learners	Describes a main takeaway or learning from the simulated discussion teaching activity cycle linked to using and/or developing PSTs’ socioemotional skills (e.g., confidence, patience).
Practice	Describes a main takeaway or learning from the simulated discussion teaching activity cycle linked to the use of the simulated discussion activity as a way to practice and gain experience for teaching real students or a way to gather information about one’s practice.
Importance of Knowing and Responding to Students	Describes a main takeaway or learning from the simulated discussion teaching activity cycle linked to the importance of knowing and/or responding to the students and their thoughts, ideas, perspectives, and/or behaviors.
Vague/Not Related	Describes a main takeaway or learning from the simulated discussion teaching activity cycle that is too vague to categorize.
None	Describes no main takeaway or learning from the simulated discussion experience.
Other	Describes a main takeaway or learning from the simulated discussion teaching activity cycle that is not one of the previous codes.
Did Not Respond	This code was applied when the preservice teacher did not provide a written response to this survey question.

Another key learning, reported from 27% of the PSTs, was the importance of being flexible when preparing for and leading a discussion, especially as a variety of different student ideas may emerge during the discussion; being willing and making space to take up and follow these student ideas is important to facilitating high-quality discussions. For example, PST 101’s main takeaway was “…to expect the unexpected. Lessons do not always carry out how you may want them to.” Some PSTs (12% of PST responses) included both aspects of the importance of preparation and the importance of being flexible in their responses. For example, PST 304’s main takeaway was, “…that it is very important to be prepared for a lesson. No matter how much you prepare, the students will most likely move this discussion in a way you did not prepare for.” Similarly, PST 102 stated, “Be more prepared and be ready to be flexible, plans change depending on children’s understanding.”

In addition, a little more than a third of the PSTs (39% of PST responses) connected their learning from the simulated teaching experience to one or more of the five key features of high-quality argumentation-focused discussion that were identified in the *Mystery Powder* science task. The PSTs reported that the simulated teaching experience helped them with ensuring all student voices are heard and ideas are valued (20% of PST responses), focusing on the accuracy of content and students’ opportunities to evaluate content accuracy (12% of PST responses), and focusing on the extent to which argument construction and critique are a focus of instruction (10% of PST responses). For example, in terms of student participation, PST 204 learned that they need to, “…work on inviting thoughts from students rather than jumping in and taking the lead…”. Furthermore, in terms of student interactions, PST 210 learned to “have the students lead the discussion more and try to rely on yourself less.” Also, in terms of student understanding, PST 103 learned that they need to “…make sure that students fully understand why the answer is what it is. I need to further explain things in the future.”

Other codes indicating additional takeaways from the simulated teaching experience, such as recognizing the importance of this teaching practice or learning how to regulate their own socioemotional skills as learners themselves, were reported by less than 10% of PSTs. For example, PST 804 stated that, “The main takeaway that I have is how important facilitating discussions in class are.” Additionally, PST 317 thought the simulated discussion was “…a unique way of gaining some teaching experience and a good way of practicing how to lead a small-group discussion.” Table 3 shows, in summary, the main takeaways and learnings PSTs provided in their responses.

**Table 3:** Results for Perceptions of Learning from the Simulated Teaching Experience

Code	Teacher Educators (*n* = 4) n	Preservice Teachers (*n* = 49) n (%)
Related to Dimensions of Argumentation-Focused Discussions	2	19 (39%)
Student Participation (Dimension 1)	2	10 (20%)
Discussion Structure (Dimension 2)	1	3 (6%)
Student Interactions (Dimension 3)	0	4 (8%)
Student Understanding (Dimension 4)	1	6 (12%)
Argumentation (Dimension 5)	0	5 (10%)
Preparation Importance	2	15 (31%)
Flexible	1	13 (27%)
Recognize Discussion Importance/Challenge	0	3 (6%)
Preservice Teachers’ Emotional Regulation	0	3 (6%)
Practice	0	3 (6%)
Knowing and Responding to Students	2	2 (4%)
Vague	1	2 (4%)
None	0	1 (2%)
Other	0	1 (2%)
Did Not Respond	0	5 (10%)

#### Challenges with the simulated teaching experience

Sixty-five percent of PSTs (*n* = 32) agreed or somewhat agreed that they did not perform as well as they could have in the simulated classroom, and 29 of those 32 PSTs provided a written response explaining why they did not perform as well as they could have facilitating the science discussion. Table 4 shows the various codes applied to explain why some PSTs felt they did not perform as well as they could have in the simulated discussion. The coding classification revealed that the PSTs were most likely to cite two main reasons why they did not perform as well as they could have, and both these reasons were not related to the simulated environment.

**Table 4:** Coding for Preservice Teachers’ Perceptions about Discussion Performance

Code	Description for Perceptions
Student Participation (Dimension 1)	Describes not performing as well as they could have due to their responsiveness to students, their focus on making sure that the discussion is grounded in students’ ideas, and/or that all students are engaged in some meaningful component of the discussion.
Discussion Structure (Dimension 2)	Describes not performing as well as they could have due to the degree to which the teacher is able to shape a coherent discussion and focuses in particular on building and connecting ideas toward a learning goal.
Student Interactions (Dimension 3)	Describes not performing as well as they could have due to the ways they attempted to facilitate the discussion so that students are responsible for explaining key ideas.
Student Understanding (Dimension 4)	Describes not performing as well as they could have due to the extent to which teacher and students are involved in the evaluation of ideas that are put forth during the discussion and/or focusing on helping students to build their conceptual understanding.
Argumentation (Dimension 5)	Describes not performing as well as they could have due to the degree to which students engage in argumentation.
Preservice Teacher Characteristics	Describes not performing as well as they could have due to the PST’s personal characteristics.
Lack of Preparation	Describes not performing as well as they could have due to a lack of preparation.
Other	Describes not performing as well as they could have due to a reason that is not one of the previous codes.
Technology Issues	Describes not performing as well as they could have due to issues with technology and/or unfamiliarity with technology.
Context	Describes not performing as well as they could have due to the context of the simulation task being dissimilar from what would normally be found in a real classroom.
Students	Describes not performing as well as they could have due to the differences in how the students respond and/or behave.
Inexperience in Classrooms	Describes not performing as well as they could have due to the PST’s lack of familiarity with teaching in general.
Discussion Time	Describes not performing as well as they could have due to experiencing issues with the allotted discussion time.
Vague/Not Related	Describes not performing as well as they could have due to a reason that is too vague to categorize.
Did Not Respond	This code was applied when the preservice teacher did not provide a written response to this survey question.
Not Prompted to Respond	This code was applied for the preservice teachers who indicated that they thought they did perform as well as they could have in the simulated classroom, as this initial response meant they did not need to explain why they failed to perform as well as they could have in the simulated classroom.

The first reason was related to the PSTs’ own personal characteristics (22% of PSTs), such as nervousness or lack of confidence. For example, PST 108 mentioned, “My nerves got the better of me…making me less confident.” The second reason was related to specific difficulties some PSTs experienced regarding one or more of the five key features of high-quality argumentation-focused discussion identified in the *Mystery Powder* task (22% of PSTs). Specifically, these PSTs noted that they struggled with the following instructional aspects when facilitating the science discussion: ensuring all student voices are heard and ideas are valued (8% of PSTs), emphasizing the structure and clarity of what transpires in the discussion (4% of PSTs), focusing on the nature and extent of teacher mediation of student contributions (10% of PSTs), ensuring the accuracy of the content and providing students with opportunities to evaluate content accuracy (10% of PSTs), and providing opportunities for students to engage in argument construction and critique (4% of PSTs). For example, PST 204 called out their challenges with student interactions: “I did not make the discussion as student led as I wanted to. I lead the entire discussion.” Similarly, PST 812 linked their reason to difficulty engaging students in aspects of scientific argumentation: “I could have asked the students to use more of their claims. I also could have asked those students who were not correct to argue their points better.”

A small proportion of PSTs also explained how their lack of preparation (12% of PSTs) was the reason they did not perform as well as they could have. For instance, PST 809 noted, “[I was] not really prepared for the discussion. [We] spent one class going over 3 slides and then used the given materials from my email to prepare.” Aside from linking to themselves or the five key features of high-quality argumentation-focused discussion as the reason they did not perform well, PSTs also gave reasons related to various external factors that were part of the simulated discussion, although these other reasons were only mentioned by a small proportion of the PSTs. For instance, PSTs described not performing as well as they could have due to the context of the simulation task being dissimilar from what would normally be found in a real classroom (4% of PSTs), issues or unfamiliarity with technology (6% of PSTs), differences in how the avatar students respond and/or behave compared to real students (4% of PSTs), and issues with the 20-min allotted discussion time (2% of PSTs). For instance, PST 112 mentioned that the simulated discussion environment was “…a new and foreign concept that...I was not too comfortable with…”. Moreover, PST 308’s reason linked back to the avatar students’ behavior: “In a real classroom, students would be more interactive and not always wait to be told to answer a question.” PSTs also mentioned a general lack of experience with teaching as the reason for them not performing as well as they could have (4% of PSTs). Table 5 shows the reasons PSTs provided for not performing as well as they could have. Overall, most PSTs cited factors other than the simulated environment as the primary challenge, such as nervousness and difficulties engaging in specific teaching moves, which are ones that you would expect to see for any teaching experience that PSTs engage in.

**Table 5:** Results for Preservice Teachers’ Perceptions about Discussion Performance

Code	Preservice Teachers (n = 49) n (%)
Related to Dimensions of Argumentation-Focused Discussions	11 (22%)
Student Participation (Dimension 1)	4 (8%)
Discussion Structure (Dimension 2)	2 (4%)
Student Interactions (Dimension 3)	5 (10%)
Student Understanding (Dimension 4)	5 (10%)
Argumentation (Dimension 5)	2 (4%)
Preservice Teacher Characteristics	11 (22%)
Lack of Preparation	6 (12%)
Other	4 (8%)
Technology Issues	3 (6%)
Context	2 (4%)
Students	2 (4%)
Inexperience in Classrooms	2 (4%)
Discussion Time	1 (2%)
Vague/Not Related	1 (2%)
Did Not Respond	3 (6%)
Not Prompted to Respond	17 (35%)

While we had 49 elementary science preservice teachers (PSTs) participate in this study, 32 of them (65%) agreed or somewhat agreed that they did not perform as well as they could have in the simulated classroom, and therefore were prompted to provide a written response explaining why. Of those 32 PSTs, 29 of them provided a written response explaining why they did not perform as well as they could have facilitating the science discussion. Here we keep the total number of PSTs as 49 PSTs, since that was the number of PSTs who originally responded to the question about whether they performed as well as they could have in the simulated classroom

### TEs’ perspectives on integrating simulated teaching experiences within elementary methods courses (TE integration)

#### Main affordances

The TEs’ perspectives on the affordances of using the simulated teaching experience within elementary methods courses included several key ideas. One of the main patterns that emerged from the TEs’ responses was that this experience provided the PSTs with practice teaching opportunities that they had limited or no access to this semester due to the COVID-19 pandemic. In many cases the simulated teaching experience directly replaced the PSTs’ canceled field experience and provided them with their only teaching experience this semester. As noted by TE03, “…at least gave them an opportunity to, as others said, with the avatars, but it gave them a hand [sic] of what it really would be like to teach. And some of them…had told me, they hadn’t taught anybody. This was a first-time experience for them.” Similarly, TE02 explained how:

…one of the real positive things I've heard from all of my students is that they appreciate that they were able to engage in experiential learning in the course this… semester…in spite of the pandemic and not being able to go into classrooms. And they really appreciate that there were ways for them to still have those experiences, which they clearly value and think is important to their development.

This opportunity to “get a feel again for the elementary classroom” (TE08) after being away from in-person classroom instruction was valued by all four TEs. Complementary to this idea is the notion that a practice space like this provides a safe space where mistakes can be made without any consequence to real students. As TE08 noted:

…because it gives them a safe space. They're always worried they're going to mess up with a kid. I'm going to mess up. I'm going to give this kid the wrong idea and I've ruined their lives. This gives them this really safe space. It's like, it's fine if you mess up. These are actors. Who cares? Try it out, see what happens. You're not going to mess anything up.

Other affordances of the simulated teaching experience noted by the TEs included the opportunity to reduce complexity as well as the ability to use the video records to support targeted observation, reflection, and analysis – both by the TEs and the PSTs. The reduction of complexity occurred by focusing the PSTs’ learning on small group, as opposed to whole group, discussions. TE08 concisely summarized this benefit by stating:

They also really valued, which I didn't expect this, they really valued being able to work with a small group. I actually thought that they would complain about that because they wanted to do a whole class discussion. But many of them were like, “No. I really understood now the learning. If I get a small group of kids together and we focus on an idea ... And I can really get in depth into their thinking.” So they really valued that, which totally surprised me.

This perspective points to how the simulated teaching experience can easily be tailored to address specific learning goals and zoom in on specific aspects or features of instruction, while limiting others that may distract from PST learning.

In addition, the simulated teaching experience, particularly the provision of the video record of the discussion in the simulated classroom, allowed the TEs to observe what the PSTs had done (as opposed to what the PSTs elected not to do) and to make their own judgments about what they had done well and identify their areas of growth. While most TEs spoke about the class of PSTs in broad terms and reflected patterns of strength or weakness, a few exceptions stood out. For example, TE02 noted ‘a wide range of students’, suggesting that the videos allowed him to see variability in his PSTs’ skills while TE03 noted that what she saw informed what she did for the remainder of the semester, as it gave her information about what they needed more support on moving forward. Similarly, TE08 made use of the videos programmatically, to inform decisions about individual student teaching placements based on observed strengths and weaknesses, and to inform reflection about where the program as a whole might or might not be with providing certain supports. TE08 similarly noted things that she would have been better prepared to observe had she not lost the opportunity to observe live instruction during the semester, such as how the PSTs “take that learning [from their methods course] and start building on that with live children in a classroom,” driving home how much was lost from a semester with no access to field placements in elementary classrooms.

#### Key challenges

The main challenge noted by these TEs was figuring out how to integrate the simulated teaching experience as part of their existing elementary science methods course, especially due to limited time and space in their current course. For some TEs, this challenge was even more daunting due to the additional difficulty of figuring out how to address new science content that had not previously been a part of their course. For example, TE01 commented how her challenge was to:

…be able to think through the process of what activity to put first, how to build up and scaffold the content, and what would that look like in the integration of science content? I personally had some internal conflict of how do I [sic] incorporate my science content that I've taught for many years and the immersion of…CER [claims, evidence, and reasoning] and argumentation.

Likewise, TE08 had to consider how to restructure her course, including addressing a more explicit focus on scientific argumentation and explained that her:

…challenge was the CER stuff, finding readings since I don't typically use those, but it also opened up some avenues for me. Like that was the first time I dug into the What's Your Evidence book, and I was like, “Oh, this is valuable. Maybe I should have been using this all along.”

Figuring out how to fit the simulated teaching experience into a larger course progression in a way that productively scaffolds and sequences learning activities throughout the full course and creates a coherent learning experience for the PSTs was both important and challenging for these TEs.

#### Future use beyond COVID

Four key themes surfaced in these TEs’ responses regarding their perceptions of using the simulated teaching experience post-pandemic. Most of these responses directly target what these TEs perceive as benefits of the simulated teaching experience that can continue in future use of this tool, while one primarily targets a specific challenge they perceive in continuing the use of this tool moving forward. First, two TEs discussed how the simulated classroom provides a safe space for PSTs to practice their teaching skills and learn from their experience. They felt this was especially important for novices who are “just embarking on this career” (TE01) and learning how to engage in complex teaching practices. TE08 explained how the PSTs are “always worried they’re going to mess up with a kid” but that the simulated classroom provides them with a safe space to practice and make mistakes without causing any harm to real students. Similarly, TE01 commented that:

…what we need for these particular students is for them to work in that type of environment where it's okay to make a mistake. We're all human, and that's something I think that they feel is that the students are so fragile that they're working with, they don't want to break.

The simulated teaching experience provides the PSTs with a space where they can try out novel teaching practices, make mistakes, get feedback, and try again – all without causing any harm or risk to real students.

Second, these TEs noted that the simulated teaching experience is a different kind of experience that can support PSTs in developing their teaching skills, including their ability to teach via online platforms, through experiential and interactive learning opportunities. For example, TE01 explained how “it gives the elevated and experiential opportunity to engage themselves, to have a better reflection of how they are now.” For some TEs, these types of experiential opportunities were especially important during the pandemic as the PSTs’ field experiences were limited or canceled. But thinking forward to post-COVID, some TEs remarked how this platform provides a way for the PSTs to practice interacting with students and, for one TE, gives the PSTs a place to practice the science teaching skills they learn about in their methods course, even though science is not a priority in many elementary classrooms. One TE noted how using the simulated classroom has the potential to help PSTs practice teaching online, which is one practice that may continue – at least to some extent – post-COVID.

Third, some TEs mentioned how they valued the ways in which the semi-standardized nature of the experience gave them better insight into what challenges their PSTs were encountering, which was one reason why they saw a future use of this tool post-pandemic. TE02’s response explains why she valued the “uniformity of experience” provided by the simulated teaching experience:

So if I send them out to classrooms, they're going to go to multiple classrooms, even if I send them in partners so there's some confirmation of what they're looking at. They're all going to see very different things. They're going to see different groups of students. They're going to see different teaching styles, all the different ways that a classroom will vary. And I think the fact that they're all having the same experience gives us a really valuable tool for... It eliminates a lot of the variables that they experience. So that's one reason I would like to really incorporate it moving forward.

The simulated teaching experience essentially reduces some of the variability that the PSTs experience across sites when in the field. This characteristic of the simulated teaching experience provides a way for TEs to get insight into how their PSTs’ teaching skills are similar or vary in response to the same teaching challenges and also allows them to better scaffold and structure future learning opportunities for their PSTs based on this comparable information. Such insights are more difficult to achieve when the PSTs are all teaching in different classrooms – and many times using different curriculums and addressing different student learning goals – as part of their field experiences.

Finally, while all TEs noted specific benefits of this experience as reasons for why they would want to continue using simulated teaching experiences in future methods course, many TEs also noted concerns they had with the expense of providing these kinds of learning opportunities for their PSTs and the time required to plan and use them within their science methods course. TE01 captured this sentiment of wanting to continue, but needed funding to do so: “And so I would definitely love if we had the funding to be able to incorporate…the Mursion simulation and then build from that experience of what to, what not to do, how to approach the methodology and pedagogy of teaching in a classroom setting.” They also expressed frustration with not knowing where to start in terms of getting access to this technology after this research project. Some TEs mentioned being hopeful that they could find the funding at their universities to continue to incorporate these simulations into their courses. However, some were not sure if their institutions could afford simulations, especially at this time of funding deficits due to the recent pandemic. Overall, there was a sense that using simulated teaching experiences in the future would require a broader institutional commitment.

## Discussion

This study’s focus was on providing an approach where PSTs could engage in some form of practice-based teaching amid a pandemic where teacher education programs were scrambling to replace field placement experiences. Findings indicated both affordances and challenges of incorporating simulated teaching experiences into online or hybrid elementary science methods courses. This is consistent with previous research in this area examining the use of such tools in face-to-face educational courses (Mikeska & Howell, 2021b; Straub et al., 2015). These findings are also consistent with the current literature on practice-based teacher education and the importance of providing PSTs with opportunities to engage in approximations of practice to support their learning (Arias & Davis, 2017; Benedict-Chambers et al., 2017; Davis, 2019; Masters, 2020).

In terms of affordances, participants were most likely to note how the simulated teaching experience provided an experiential opportunity and safe space to learn, supported the development of teaching skills, supported transfer to the classroom, and provided flexible opportunities for standardization and formative assessment. However, despite the TEs’ consistent enthusiasm about the use of simulations as a practice space and viable pedagogical approach moving forward, they also voiced concerns related to the challenges of using simulated teaching experiences as part of teacher education courses in the future. These challenges were far more practical and related primarily to the cost of simulation implementation and how to best incorporate such tools within already full elementary science methods courses. PSTs also expressed concerns about their lack of preparation, lack of confidence, and limited familiarity with the simulated classroom environment, although these concerns tended to be limited to a smaller number of PSTs in comparison with the benefits noted by these study participants. Collectively, these findings suggest four important considerations.

First, findings suggest that simulated teaching experiences have the potential to support the bridging of the space between academic learning in university coursework and practical application in fieldwork that takes place in classroom-based settings. Helping PSTs make such connections has been a perennial challenge in teacher education (Allen & Wright, 2014; Korthagen et al., 2006). During the pandemic the challenge was acute, and often PSTs simply had no access to field placement. As noted in the literature, however, access to field placements is not the only challenge. Quality of mentorship in field placements is variable and impactful on what PSTs learn (Goldhaber et al., 2020; Greenberg et al., 2011). Connecting coursework and field experience can be hindered by both substantive challenges including a lack of shared vision around what good instruction is and practical challenges, such as course instructors not having information about what is happening in field placements (Zeichner & Bier, 2017). Simulation, while limited in comparison to fieldwork in some ways, offers the TE an unprecedented degree of control over and access to the PSTs’ learning experiences. It also offers a way to purposefully structure learning opportunities and provide PSTs with targeted feedback to support them in developing their teaching practice.

Second, many of the PSTs’ reports about their main learnings focused on the nature of the work of teaching and what it demands of the teacher. The challenges faced by elementary PSTs as they learn to engage in high-quality science instruction have been well documented in the current literature (Davis et al., 2006; Yoon et al., 2012). Previous literature has suggested that PSTs not only need to develop their ability to engage in critical instructional practices, but they also need to develop the beliefs, orientations, identities, and understandings to support their instructional decision-making (Chen & Mensah, 2018; Kurup et al., 2019; Menon & Sadler, 2016). This study’s findings suggest that, in addition to the obvious ‘learn by practice’ mechanism, there might be another learning mechanism at play by which PSTs’ vision of what high-quality science instruction is may be impacted by using such tools. This is possibly an underexplored mechanism by which simulation can impact PST learning that could be studied more, as the theories of action used in the field generally place simulations in the role of “approximation” and not in the role of “representation.” Understanding better how representations, decompositions, and approximations overlap within the context of online simulations and how they can be coordinated to support PST learning would be an important future contribution to theory (Grossman et al., 2009; Mikeska, Howell, Dieker, & Hynes, 2021).

Third, this study offers emergent evidence that simulation approaches are flexible enough to be used under varying course formats. To our knowledge, course format is not a variable that has been explored in detail in prior literature on simulation use, likely because hybrid courses were less commonly used in teacher preparation prior to the COVID-19 pandemic in comparison to in-person courses. In fact, prior to 2020 the technology provider we worked with, Mursion®, did not have an online-delivery compatible platform available in the education space, and simulation using this technological approach was previously conducted in-person in learning-lab environments. Others in the field have argued for the many advantages of using simulations, like the one used in this study, to support teacher learning, arguing that such tools can be used in flexible ways to address a range of learning objectives across grade levels, content areas, and student populations, which provides a way for PSTs to practice in (simulated) environments that have more variability than those the PSTs have opportunities to experience in real life (Dieker et al., 2014; Kaufman & Ireland, 2016). Moving forward, we suspect that schools of teacher education may continue to employ such formats to some degree, after having been forced to grapple more extensively with the challenges of hybrid learning environments.

Finally, study findings point to the importance of providing appropriate supports to help PSTs learn to prepare for and engage in productive instructional decision-making during their lesson planning and enactment. Study findings demonstrated that the simulated teaching experience helped the PSTs recognize the importance of adequately planning to facilitate these discussions and how to ensure that they could flexibility attend to the students’ ideas during the discussions. Previous research has also noted the importance of helping teachers build their knowledge and ability to effectively plan, including how to critically evaluate potential resources for use in this process, and to engage in responsive science teaching (Colley & Windschitl, 2016; Janssen et al., 2019; Kang, 2017; Robertson et al., 2016; Sawyer et al., 2020). These findings suggest that simulated teaching experiences could be one tool that can help develop PSTs’ abilities in these pedagogical areas.

Based on this study’s findings, we argue that using simulated teaching experiences is one technologically based approach that science teacher educators should consider incorporating into their instructional repertoire to develop elementary science PSTs’ instructional capabilities. Such experiences can provide practice-based teaching opportunities to support PST development of ambitious and complex core teaching practices, such as learning how to facilitate argumentation-focused discussions. These experiences can help provide learning opportunities for PSTs in situations where access to high-quality K-12 field experiences is limited, such as the conditions under which this study was conducted. But they can also provide richer learning opportunities even in cases where access to field experiences is not problematic, by ensuring PSTs face key challenges that might or might not come up in naturalistic settings and receive strong mentorship from a TE which may or may not be available through a field placement.

## Limitations

This study has three main limitations. First, this study relied primarily on self-report data from study participants to understand their perceptions of affordances, challenges, and opportunities for using simulated teaching experiences. Since study results target PSTs’ and TEs’ perceptions about the affordances and challenges of using this tool to support PST learning, we are unable to draw strong conclusions about how the simulated teaching experience impacted changes to PSTs’ practice, knowledge, or beliefs. However, other studies using similar tools have shown evidence of PSTs’ improvement in their ability to engage in this core teaching practice (Mikeska & Howell, 2021b; Straub et al., 2015), suggesting that such use is linked to PST learning.

Second, one limitation is concerned with the possibility for participants to provide socially desirable responses on their perceptions of the simulated teaching experience. However, this limitation was mitigated with explicit framing in the survey and interview instruments where our research team explained the study’s purpose to seek out feedback to understand the full complexity, potential, and pitfalls with the simulated teaching experience. Study findings indicate that TEs and PSTs openly shared affordances and substantive critiques of using simulated teaching experiences, suggesting that this limitation was mitigated.

Finally, this study oversampled minority serving institutions and selected participants with the direct goal of addressing COVID-19 induced challenges to instruction. This resulted in a PST sample that is somewhat more diverse than the national population of elementary science PSTs and a TE population that was participating under unusually difficult conditions. As such, results cannot be used to generalize to the population of elementary science TEs and elementary PSTs across the United States or to more traditional semester implementations.

## Conclusion

Overall, this research study contributes to the nascent research on the possibilities for leveraging technologies, in this case simulated classroom environments, to productively develop elementary science PSTs’ competencies both during and after the COVID-19 pandemic in the context of fully online or hybrid courses. Collectively, study findings suggest cautious optimism for using simulated teaching experiences to support PSTs in learning how to facilitate argumentation-focused science discussions within online or hybrid method courses. In general, TEs’ responses to the use of the simulated teaching experience was overwhelmingly positive despite challenges they encountered implementing the new approach for the first time. PSTs, on the other hand, showed more mixed reactions, with most reporting learning in ways that were consistent with the task design and the TEs’ instructional goals, but with a notable minority reporting challenges related to the technology itself. In conclusion, we point to two areas in which we see ways to improve the approach, but which also point to areas in which the field might consider more dramatic shifts to capacity and infrastructure.

Study findings suggest that future development and research efforts in this area should more explicitly attend to the need for structured and guided supports for TEs in how to integrate simulated teaching experiences into their elementary science methods courses. However, additional guidance does not create more time. That the TEs reported a real tension fitting an activity they found deeply worthwhile into an already crowded course syllabi points to the reality of having relatively few hours of instructional time in most programs devoted to foundational and critical teaching practice. A true solution goes beyond the scope of this project and might require a more substantial commitment to allocating sufficient time in methods courses to address these goals.

In addition, an institutional commitment is likely also the practical answer to cost considerations. While reducing costs for simulation is a worthwhile goal, the cost of an approach reliant on trained interactors likely has a minimal threshold. A growing number of institutions do manage this challenge effectively at a programmatic level by making a commitment to supporting simulation use similar to the commitment they might make through laboratory or facility investments in other programs. Generating and providing TEs with strategies for garnering and maintaining such support at an institutional level would be useful. Doing so would help to better ensure the viability of this practice-based approach to be sustained over time.

## Implications

The study’s findings suggest there are important challenges that require attention for such tools to be used productively and made available within a diversity of teacher education settings. First, TEs would benefit from additional supports so they can help their PSTs prepare for their simulated teaching experience. For some of these TEs, the focus on scientific argumentation and how to help PSTs learn to support that in science discussions was a novel one in their course. Research that provides concrete examples and tools for TEs to use as they scaffold their PSTs in learning how to engage in this ambitious teaching practice within the context of a simulated classroom would be an important contribution.

Second, PSTs also may need more direct, scaffolded support in figuring out how to plan for the simulated discussion. This support may focus on the task’s science content or how to engage in specific teaching moves, such as eliciting and connecting students’ ideas, shaping a coherent discussion, and encouraging students to build towards consensus. This support also may include work to help PSTs understand what it means to plan adequately and support in knowing what it is they should be planning to do. Some ideas include providing video-based examples of other teachers engaged in this core teaching practice for PSTs to analyze and decompose and having PSTs co-plan with one another with feedback from their peers and TE.

Optimal use of simulations likely also requires careful attention to structuring PSTs’ opportunities to learn from reflection and helping them develop the appropriate skills to make sense of and accurately assess their own teaching (or that of others). Structured reflection could also be coupled with repeated simulated teaching experiences where the PSTs could continue to refine and apply their newly learned knowledge and skills. In addition, increasing PSTs’ understanding of the features of high-quality science discussions and their familiarization of the simulation classroom would also likely help to decrease their nervousness about the experience. For example, TEs could have the PSTs watch videos of the student avatars talking to one another, have them engage in a ‘meet and greet’ with the avatars ahead of time, and practice warm up tasks (e.g., talking to the avatars about their hobbies) unrelated to the core teaching practice. Alternatively, TEs could model the use of the simulated classroom with their PSTs to provide them with opportunities to become familiar with this tool and see it in action.

Finally, a more practical need is in helping individual TEs and teacher education programs consider how to provide access to the simulated teaching experience considering the tool’s expense. While research grants can support such access, it would be useful for the field to also consider the development of an open-source simulated classroom to be used more broadly across programs. Some development has begun in this area with the recent testing of an online virtual teaching simulator for use in teacher education programs showing promise (Mikeska, Shekell, et al., 2022).

## Data Availability

Data used in this study may be accessed through a written request to the project’s principal investigator (Jamie N. Mikeska) at jmikeska@ets.org or Educational Testing Service, 660 Rosedale Rd., Princeton, NJ 08541.

## Footnotes

1 The larger research study included elementary teacher educators in two content areas (mathematics and science) and our total recruitment efforts reflect elementary teacher educators across both areas. In our larger study, we selected a total of eight teacher educators – four in elementary science and four in elementary mathematics – for participation. In our selection across the full set of eight teacher educators, we also attended to the balance of institution type and geographical location. This manuscript analyzes and reports on the findings for the four elementary science teacher educators and their preservice teachers.

2 The Argumentation Toolkit was funded by the National Science Foundation (grant #1119584) and the online tools and videos are available at: http://www.argumentationtoolkit.org/intro.html.
